# A Lightweight Vision-Language-Action Policy with Progress-Aware Hybrid Execution for UAV Waypoint Navigation in AirSim

**DOI:** 10.3390/s26144655

**Published:** 2026-07-22

**Authors:** Yiqing Xu, Haifeng Lin, Yujin Yang, Ji’An Xia, Zidong Han

**Affiliations:** 1 School of Computer and Software/School of Artificial Intelligence, Nanjing University of Industry Technology, Nanjing 210023, China; 2019101041@niit.edu.cn; 2 College of Information Science and Technology & Artificial Intelligence, Nanjing Forestry University, Nanjing 210037, China; haifeng.lin@njfu.edu.cn; 3 School of Transportation Engineering, Nanjing University of Industry Technology, Nanjing 210023, China; yujin.yang@niit.edu.cn; 4 School of Aeronautic Engineering, Nanjing University of Industry Technology, Nanjing 210023, China; siton.han@nuaa.edu.cn

**Keywords:** UAV navigation, vision-language-action model, RGB-D perception, AirSim, imitation learning, hybrid execution, lightweight neural networks

## Abstract

Offline action prediction does not by itself guarantee reliable closed-loop flight for unmanned aerial vehicle (UAV) vision-language-action (VLA) models. We study a controlled AirSim Blocks waypoint task using 100 expert episodes and 3385 RGB-D, instruction, state, and action records. Checkpoints are selected only on val-seen data, after which val-unseen is evaluated once. A 132,840-parameter policy reaches 0.9278±0.0019 final-test action accuracy across three training seeds, yet raw VLA control fails in closed loop. We therefore embed its action proposals in progress-aware hybrid execution with explicit recovery and near-goal precision. The strongest checkpoint reaches 59/60 goals, but crossing three independently trained checkpoints with three target seeds yields a more conservative 147/180 successes (81.7%) with zero recorded collisions and marked checkpoint sensitivity. Substantial overrides and fallback-tagged steps further show that the reported closed-loop outcomes are properties of the hybrid system, not of the learned policy alone. These findings are restricted to the controlled AirSim Blocks benchmark and do not demonstrate real-UAV deployment, sim-to-real transfer, or field robustness.

## 1. Introduction

Autonomous unmanned aerial vehicles (UAVs) are increasingly expected to operate in settings where navigation objectives may be specified through a mixture of coordinates, visual context, and language-like instructions. Even in a controlled waypoint task, the system must connect visual perception, instruction-conditioned action selection, and low-level flight execution. This makes UAV navigation a useful testbed for studying vision-language-action (VLA) models, drawing on progress in embodied navigation [1,2] and robotic VLA systems [3,4]. It also makes overclaiming easy: a policy that predicts local action labels from simulator state is not automatically a robust language-grounded aerial robot.

However, UAV-VLA differs substantially from VLA for tabletop manipulation or slow ground-robot navigation. First, aerial platforms are highly sensitive to short sequences of erroneous actions: a small yaw error, an unnecessary vertical command, or a premature stop can rapidly accumulate into large spatial deviation. Second, UAV control is inherently closed-loop. A policy that predicts plausible action labels offline may still fail when its own actions change the future observation distribution. Third, real UAV experiments introduce hardware cost, safety risk, battery constraints, and site limitations, which make broad reproduction difficult. These factors create a practical gap between large, system-level VLA demonstrations and the needs of low-cost, hardware-free UAV research.

This paper studies a deliberately focused version of the UAV-VLA problem. Rather than attempting to deploy a large end-to-end VLA model on a real drone, we ask whether a compact action-level RGB-D VLA policy can be trained, evaluated, and diagnosed in a fully traceable AirSim pipeline [5]. The environment is AirSim Blocks, the language is templated, the task is waypoint-oriented, and the goal is to quantify what can and cannot be concluded from a low-cost UAV-VLA experiment. The motivation is first-principled: before claiming full real-world UAV autonomy, one should verify whether the multimodal action module benefits from vision, language, state, and action history, whether geometric shortcuts dominate the result, and whether the resulting policy remains reliable when placed in closed-loop control.

To this end, we construct an AirSim RGB-D VLA Blocks pilot dataset. Each step contains a front RGB image, depth observation, natural-language instruction, UAV state vector, previous action, and expert action label. The dataset supports controlled ablations over state-only, vision-only, language-vision, and full VLA input configurations. It also supports closed-loop evaluation in the same AirSim Blocks environment, allowing the raw learned policy to be compared with progressively stronger hybrid execution variants.

The central empirical finding is that lightweight multimodal action imitation is feasible but insufficient by itself. A compact VLA can learn the teacher-forced action labels in this controlled setting, and diagnostic ablations help separate useful multimodal input from geometric shortcuts. Yet the raw policy remains fragile in closed-loop deployment because a step-level classifier can learn expert labels while still lacking the progress monitoring, recovery behavior, and near-target precision needed for reliable autonomous navigation.

We therefore introduce and evaluate progress-aware hybrid execution around the learned VLA policy. The action governor monitors whether the UAV is making progress toward the target and replaces unsafe or non-progressing actions with geometry-based recovery primitives. A near-goal precision controller reduces aggressive motion close to the stopping threshold, mitigating overshoot and unstable termination. The correct interpretation is not that raw VLA solves UAV navigation, but that a lightweight VLA can become useful when embedded in an explicit, measurable execution layer.

The contributions of this work are as follows:

- We build a low-cost, hardware-free, and traceable AirSim RGB-D UAV-VLA pilot dataset containing synchronized RGB, depth, language, state, previous-action, and expert-action fields.
- We design and evaluate a tiny RGB-D VLA action policy that fuses visual observations, natural-language instruction, UAV state, and previous action, while keeping the model small enough for fast action-level inference.
- We add no-coordinate language and no-goal-state ablations to test whether the result depends on explicit coordinate tokens or geometric state shortcuts. These ablations show that instruction coordinates are not the main driver, but explicit goal geometry still contributes substantially.
- We show through a locked-checkpoint execution ablation and a three-checkpoint by three-target-seed closed-loop evaluation that raw VLA action prediction is not sufficient for UAV navigation. Progress-aware hybrid execution and precision control are necessary for reliable AirSim navigation, while the crossed evaluation exposes substantial training-seed sensitivity.
- We provide a careful efficiency and evidence-bound analysis: the full visual VLA contains 132,840 parameters, occupies 0.521 MB, and runs at 0.9347 ms per CPU step, but this action-level tiny VLA should not be directly compared with large token-level VLA inference or real-drone deployment systems.

The rest of the paper is organized as follows. Section 2 reviews UAV vision-language navigation, VLA models, hybrid execution, and lightweight deployment. Section 3 describes the AirSim RGB-D VLA benchmark, model variants, and execution module. Section 4 reports offline ablations, closed-loop comparisons, robustness analysis, and efficiency results. Section 5 discusses validity threats, scope, and future validation. Section 6 concludes with limitations and future directions.

## 2. Related Work

### 2.1. UAV Vision-and-Language Navigation

Vision-and-language navigation has traditionally focused on ground-level agents that follow natural-language instructions through indoor environments, beginning with the Room-to-Room task and speaker-follower training setup [1,6]. Later VLN agents introduced progress estimation and pretraining to improve generalization and route completion [7,8]. Recurrent cross-modal BERT encoders, history-aware transformers, and dual-scale graph reasoning further expanded the model family for navigation instruction following [9,10,11]. Outdoor and street-view navigation datasets extend this line toward spatial reasoning at urban scale [12,13]. UAV navigation changes the problem geometry and the failure modes. Aerial agents operate in three-dimensional space, must handle altitude and yaw control, and often require long-range semantic reasoning over sparse visual cues. UAV-oriented vision-and-language navigation benchmarks have therefore introduced aerial trajectories, panoramic or front-view observations, and metrics such as navigation error, success rate, oracle success rate, and path efficiency [2]. More recent peer-reviewed work expands this setting toward real-city aerial data, direction-aware language grounding, and tiered hybrid planning [14,15,16]. These benchmarks show that aerial VLN remains difficult: route-level semantic reasoning, stopping accuracy, unseen-scene transfer, and efficient execution remain coupled challenges.

This literature motivates two observations that are central to our work. First, language alone is insufficient for low-level UAV control, because the same high-level instruction may correspond to many different step-wise actions depending on state, heading, depth, and goal geometry. Second, successful aerial navigation usually requires a hierarchical structure: semantic interpretation and target selection at a higher level, followed by a reliable local execution mechanism. Our preliminary AerialVLN-S experiments support the same view. Temporal state history provides strong offline action prediction, while language by itself performs poorly for step-level action classification. We therefore use UAV-VLN results as contextual evidence, but we do not directly compare our AirSim Blocks action accuracy with published SR, SPL, NE, or SDTW numbers, because the task definitions and evaluation protocols are different.

### 2.2. Vision-Language-Action Models for Robotic Control

Vision-language-action models extend vision-language representation learning toward embodied control by predicting robot actions from visual observations and language instructions. RT-1 and RT-2 demonstrate large-scale robotic transformer policies and web-knowledge transfer to control [3,17]. Open X-Embodiment, OpenVLA, and Octo show how broader robot datasets and open generalist policies can support cross-embodiment transfer and adaptation [4,18,19]. Other embodied language systems ground high-level instructions in robot affordances or multimodal planning rather than directly claiming low-level flight control [20,21]. Imitation-learning policies such as BC-Z, diffusion policies, and action-chunking transformers further illustrate the range of action representations used in robot learning [22,23,24]. These models highlight the promise of unifying perception, instruction following, and control, but they also expose a deployment tension: high-capacity models are expensive to train and run, while many robotic platforms require fast, bounded, and verifiable action execution.

For UAVs, this tension is sharper. The action space is not simply a sequence of symbolic decisions; predicted actions must be converted into flight commands under stability, collision, altitude, and stopping constraints. Recent aerial VLA preprints explore large-scale mission generation, onboard geometric safety correction, and minimalist end-to-end control [25,26]. We cite these works as current context rather than peer-reviewed baselines. Their designs reinforce the need to distinguish a learned multimodal proposal from the downstream action interface or controller. Our study instead focuses on an extremely lightweight, action-level RGB-D VLA under a fully hardware-free AirSim setting. The goal is not to match the scale or task breadth of recent aerial VLA systems, but to isolate when compact multimodal action imitation helps and when explicit execution logic is still required.

### 2.3. Hybrid Execution and Closed-Loop Control

Closed-loop navigation exposes errors that are invisible in offline classification. A policy can achieve high validation accuracy on expert labels while still drifting, oscillating, stopping too early, or failing to recover after an incorrect command. This problem is particularly severe for UAVs because action errors can rapidly change viewpoint, altitude, and future observations. Progress-monitoring and regretful VLN agents already show the value of estimating whether navigation is actually moving toward the goal [7,27]. Imitation-learning theory gives a complementary warning: supervised policies can fail under the state distribution induced by their own actions [28]. Safety-critical control and local collision-avoidance methods provide another relevant layer for constraining or replacing unsafe learned actions [29,30]. For aerial systems, trajectory generation and flight-control middleware also make clear that learned decisions usually need a lower-level execution interface rather than direct unbounded actuation [31,32].

Hybrid execution can take several forms: obstacle-distance checks, action override rules, progress monitoring, speed reduction near the target, waypoint tracking, and fallback controllers when the learned policy fails to reduce distance to the goal. In our setting, this distinction is architectural rather than cosmetic. The learned policy proposes an action primitive, but the executable UAV command is produced only after the proposal has passed through transparent progress and safety checks. This design makes the final success rate attributable to a hybrid system rather than to raw VLA inference alone.

### 2.4. Lightweight and Hardware-Free UAV Learning

Many UAV learning systems require either real flight data, large simulator assets, or powerful onboard compute. Such requirements can be prohibitive for early-stage research, where the goal is often to test architectural hypotheses rather than to build a complete field-ready platform. Hardware-free simulation provides a practical intermediate layer: it cannot replace real-world validation, but it can reveal failure modes, support controlled ablations, and make reproduction easier under limited resources. AirSim and Flightmare are especially relevant for aerial or autonomous-vehicle simulation [5,33]. CARLA and Habitat illustrate the broader role of reproducible simulators in autonomous driving and embodied-AI navigation [34,35]. Gibson and AI2-THOR provide complementary examples of interactive 3D environments for perception and visual AI research [36,37].

Lightweight models are also important for UAV control. Even when a high-level planner is available, the local action module may need to run at high frequency on a low-power processor. Compact visual backbones such as MobileNets, SqueezeNet, and EfficientNet show the long-standing value of small perception models for resource-constrained settings [38,39,40]. At the same time, transformer, BERT, and CLIP-style pretraining established the representation-learning backdrop for many modern vision-language systems [41,42,43]. For real UAV deployment, onboard autonomy platforms such as PIXHAWK highlight why compute and integration constraints matter in practice [44]. A compact policy with a small memory footprint is easier to deploy, profile, and debug than a large token-level model. Our full visual VLA has only 132,840 parameters and a 0.521 MB checkpoint, and its batch-one CPU latency is below 1 ms in our profiling. This does not imply equivalence to large VLA inference or real onboard deployment, since our model is an action-level tiny network evaluated in AirSim. It does, however, establish a useful lower-cost route for studying RGB-D VLA action prediction and hybrid execution before moving to larger-scale datasets or real UAV hardware.

### 2.5. Positioning of This Work

The above strands suggest a consistent picture. UAV-VLN benchmarks show that aerial language navigation is difficult and benefits from hierarchy. Large VLA systems show that multimodal policies can connect perception and action, but they often require substantial data and compute. Robotics safety and local-control research shows that learned actions must be constrained and monitored in closed loop. Lightweight simulation-based research provides a low-cost route to isolate these issues before real deployment. Table 1 summarizes the positioning gap addressed by this work: the paper does not claim large-scale open-world aerial autonomy, but instead studies whether a tiny multimodal action proposer can be made useful inside an explicit progress-aware execution layer.

## 3. Method

### 3.1. Mathematical Modelling and Execution Interface

We formulate lightweight UAV-VLA navigation as action-primitive prediction followed by hybrid execution. At each time step *t*, the UAV receives a multimodal observation

(1) $$O_{t}=\left\{I,x_{t}^{rgb},x_{t}^{d},s_{t},a_{t-1}\right\},$$

where *I* is a natural-language instruction, $x_{t}^{rgb}$ is the front RGB image, $x_{t}^{d}$ is the depth observation, $s_{t}\in R^{D_{s}}$ is the UAV state vector, and $a_{t-1}$ is the previous discrete action. The learning objective is to predict an expert action primitive

(2) $${\hat{a}}_{t}=f_{\theta }\left(O_{t}\right),\;{\hat{a}}_{t}\in \left\{0,1,\ldots ,7\right\},$$

where the action set includes stopping, forward translation, yaw rotation, vertical motion, and lateral avoidance primitives. In the current AirSim Blocks dataset, the recorded expert labels are concentrated on stop, forward motion, yaw correction, and ascent; descent and lateral avoidance are retained in the execution interface but are sparsely or not triggered by the present Blocks trajectories. During closed-loop evaluation, the predicted action is not executed blindly. Instead, it is passed through an execution module

(3) $${\tilde{a}}_{t}=g\left({\hat{a}}_{t},s_{t},p^{goal},d_{t},H_{t}\right),$$

where $p^{goal}$ is the target position, $d_{t}$ is the front-depth statistic, and $H_{t}$ denotes short-term progress history. The final command ${\tilde{a}}_{t}$ is converted into an AirSim velocity or yaw-rate primitive. This separation reflects the central design principle of this paper: the VLA model predicts compact action primitives, while the execution module enforces progress and stable stopping.

**Figure 1:**
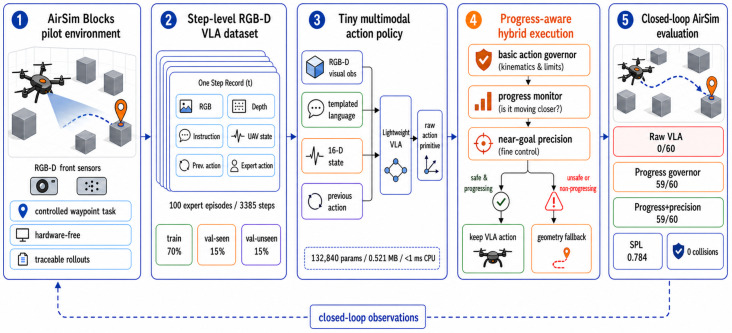
Overall workflow from the AirSim Blocks dataset to lightweight action prediction, hybrid execution, and closed-loop evaluation. All right-panel closed-loop metrics refer to the locked seed-11 execution ablation.

Figure 1 summarizes the complete dataset-to-execution workflow.

### 3.2. AirSim RGB-D VLA Pilot Dataset

The pilot dataset is generated in AirSim Blocks using scripted expert rollouts. Each episode samples a target waypoint and converts it into a templated language instruction, for example, instructing the UAV to fly toward a left, right, or center target at a specified altitude and stop when close. At every step, the system records synchronized RGB and depth observations from the front camera, the current UAV state, the target-relative state vector, the previous action, and the expert action chosen by the scripted controller. This simulator-first protocol follows the same broad motivation as prior embodied-AI simulators: controlled data generation and reproducible failure analysis before physical deployment [5,33,35].

The state vector concatenates pose, goal, target-relative displacement, scalar distance, front-depth statistic, current step index, and maximum episode length:

(4) $$s_{t}=[p_{t},\eta_{t},p^{goal},p^{goal}-p_{t},{∥p^{goal}-p_{t}∥}_{2},d_{t},t,T],$$

where $p_{t}$ is the UAV position, $\eta_{t}$ contains orientation terms used by the controller, $d_{t}$ is the median valid front-depth value, and *T* is the maximum rollout length. In the current dataset, this yields a 16-dimensional state vector. The three absolute target coordinates, three target-relative displacement coordinates, and scalar target distance are simulator-provided privileged goal-geometry fields. They are available by construction in this controlled benchmark, but they are not treated as quantities that an onboard perception stack would obtain without localization, goal estimation, and associated uncertainty.

The expert policy is geometry-based. It first stops when the target is sufficiently close, then checks for near frontal obstacles, altitude error, yaw error, and finally forward motion. The resulting action label is

(5) $$a_{t}=\left\{\begin{array}{cc} 0, & \mathrm{if}\;{∥p^{goal}-p_{t}∥}_{2}<\epsilon_{stop},\\ 6\;\mathrm{or}\;7, & \mathrm{if}\;\mathrm{front}\;\mathrm{depth}\;\mathrm{is}\;\mathrm{unsafe}\;\mathrm{and}\;\mathrm{lateral}\;\mathrm{avoidance}\;\mathrm{is}\;\mathrm{needed},\\ 4, & \mathrm{if}\;\mathrm{the}\;\mathrm{UAV}\;\mathrm{should}\;\mathrm{ascend},\\ 5, & \mathrm{if}\;\mathrm{the}\;\mathrm{UAV}\;\mathrm{should}\;\mathrm{descend},\\ 2\;\mathrm{or}\;3, & \mathrm{if}\;\mathrm{yaw}\;\mathrm{correction}\;\mathrm{is}\;\mathrm{required},\\ 1, & \mathrm{otherwise}. \end{array}\right.$$

This expert is intentionally simple and traceable. It is not meant to be a complete UAV planner; rather, it provides consistent step-level supervision for studying lightweight RGB-D VLA imitation under controlled conditions.

### 3.3. Tiny RGB-D VLA Policy

The proposed policy is a compact multimodal network that fuses four input streams: RGB-D visual observation, language instruction, UAV state, and previous action. It deliberately uses small learned encoders instead of importing a large pretrained backbone, because the experiment is meant to isolate action-level evidence rather than scale model capacity. Let the RGB-D tensor be

(6) $$x_{t}=\mathrm{concat}\left(x_{t}^{rgb},x_{t}^{d}\right)\in R^{4\times H\times W}.$$

The visual encoder is a small convolutional network:

(7) $$z_{t}^{vis}=E_{vis}\left(x_{t}\right),$$

consisting of three strided convolutional blocks, batch normalization, ReLU activations, adaptive average pooling, and a linear projection. This design keeps the visual path deliberately lightweight rather than relying on a large pretrained vision backbone.

The instruction is tokenized into a sequence of word indices and embedded by a learned lookup table. Given token embeddings $e_{1},\ldots ,e_{L}$, the text feature is average pooled over non-padding tokens:

(8) $$z^{txt}=\frac{1}{\max(1,L_{I})}\sum_{i=1}^{L}m_{i}e_{i},$$

where $m_{i}$ is the token mask and $L_{I}=\sum_{i}m_{i}$ is the instruction length after tokenization. The UAV state is normalized using training-set statistics and passed through a two-layer MLP:

(9) $$z_{t}^{state}=E_{state}\;\left(\left(s_{t}-\mu_{s}\right)/\sigma_{s}\right).$$

The previous action is represented by a learned embedding:

(10) $$z_{t}^{prev}=E_{act}\left(a_{t-1}+1\right),$$

where the offset reserves index zero for padding or missing previous action.

The full VLA feature is obtained by concatenation:

(11) $$z_{t}=\left[z_{t}^{vis};z^{txt};z_{t}^{state};z_{t}^{prev}\right],$$

followed by a lightweight fusion head:

(12) $$o_{t}=W_{2}\;\phi \left(\mathrm{LN}\left(W_{1}z_{t}+b_{1}\right)\right)+b_{2},\;p\left(a_{t}=k|O_{t}\right)=\mathrm{softmax}{\left(o_{t}\right)}_{k},$$

where ϕ(·) is ReLU and LN denotes layer normalization. The model is trained with cross-entropy loss:

(13) $$L_{act}=-\frac{1}{N}\sum_{n=1}^{N}\log p\left(a_{n}|O_{n}\right).$$

We evaluate both class-weighted and unweighted cross-entropy, train with AdamW, and use layer normalization in the fusion head [45,46]. Although class weighting can increase attention to minority action classes, the unweighted full VLA is used for closed-loop evaluation because it gives the strongest val-seen-selected final-test result among the original configurations. Closed-loop stability is not assumed from this offline score: three independently trained unweighted checkpoints are evaluated separately in Section 4.6. Table 2 gives the complete implemented architecture and preprocessing configuration for reproducibility.

**Figure 2:**
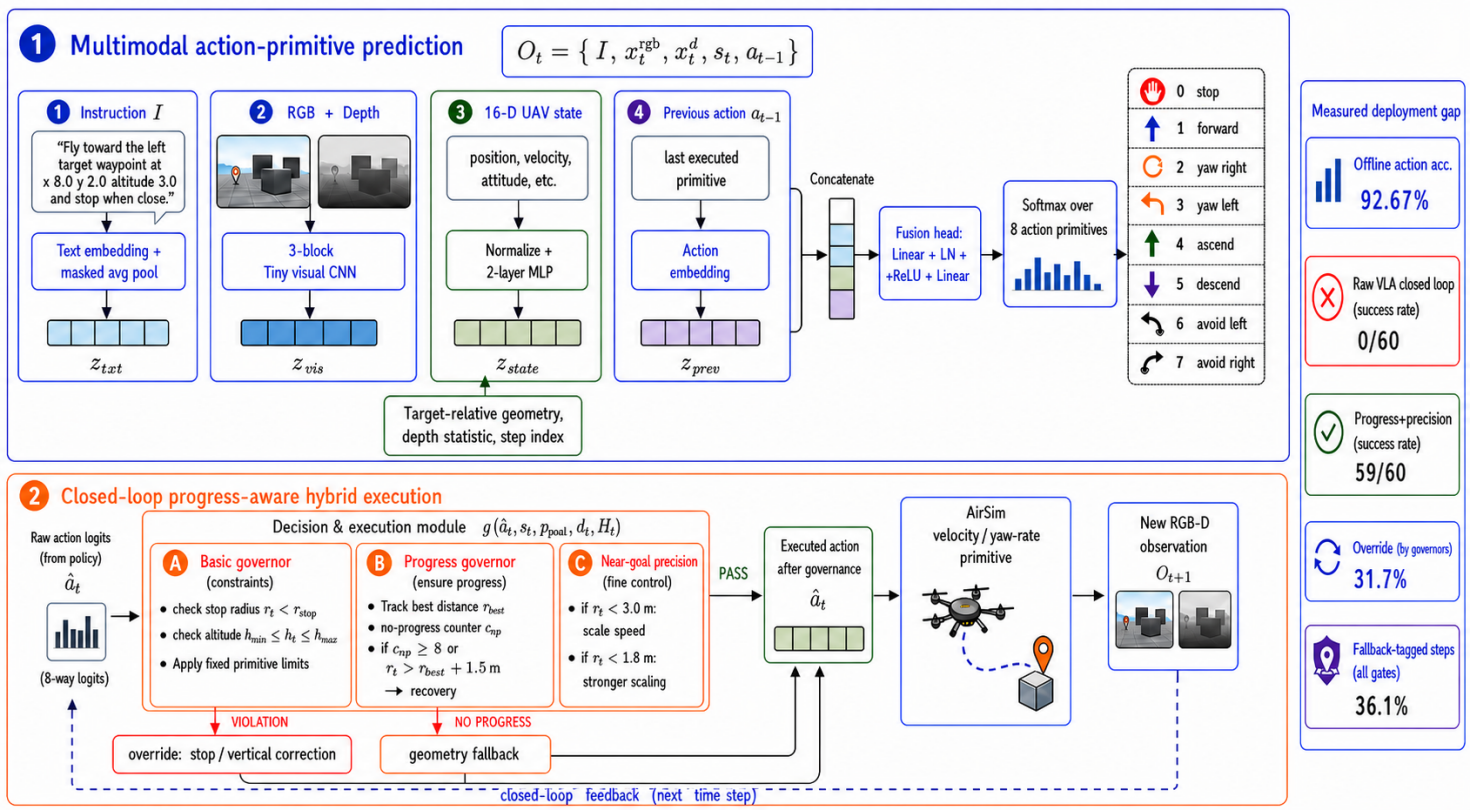
Tiny RGB-D VLA action prediction and hybrid execution interface. The diagram separates teacher-forced action imitation from the governed closed-loop command sent to AirSim; displayed metrics refer to the locked seed-11 checkpoint.

Figure 2 illustrates the separation between teacher-forced action prediction and governed closed-loop execution.

### 3.4. Modality Ablations

To isolate the contribution of each input stream, we train five variants under the same data split:

- **State-only:** uses $s_{t}$ and $a_{t-1}$ only.
- **Vision-only:** uses RGB-D observation and $a_{t-1}$ only.
- **Language + Vision:** uses instruction, RGB-D observation, and $a_{t-1}$.
- **Full VLA weighted:** uses instruction, RGB-D observation, state, and previous action with class-weighted loss.
- **Full VLA unweighted:** uses the same full input set with unweighted loss.

These ablations are important because a high score from the full model alone would not establish that multimodal fusion is useful. The controlled comparison reveals whether RGB-D, language, and state contribute complementary information in the AirSim benchmark.

We further add diagnostic controls to address shortcut concerns. In the no-coordinate language setting, numeric x/y/altitude tokens are removed from the templated instruction while preserving the directional and stopping words. In the no-goal-state setting, the state vector removes the absolute goal position, goal-relative displacement, and scalar distance, reducing the state dimension from 16 to 9. We also evaluate a no-previous-action variant that keeps RGB-D, language, and state inputs but removes the learned previous-action embedding. These variants test whether the model is merely reading coordinates from the instruction or state, or relying mostly on action-history inertia, rather than using the multimodal observation chain. A separate field audit confirms that the step records contain no next_* state field; the target-relative displacement in the state vector is goal-relative geometry, not a next-step delta.

### 3.5. Progress-Aware Hybrid Execution

The raw policy executes ${\hat{a}}_{t}$ directly. The governed policy instead applies a rule-based correction function before execution. The first layer is a basic action governor that forces a stop when the UAV is close enough to the target and replaces inconsistent vertical actions:

(14) $${\tilde{a}}_{t}=\left\{\begin{array}{cc} 0, & \mathrm{if}\;r_{t}<\epsilon_{succ},\\ a_{t}^{geo}, & \mathrm{if}\;{\hat{a}}_{t}\;\mathrm{is}\;\mathrm{inconsistent}\;\mathrm{with}\;\mathrm{altitude}\;\mathrm{error},\\ {\hat{a}}_{t}, & \mathrm{otherwise}, \end{array}\right.$$

where $r_{t}={∥p^{goal}-p_{t}∥}_{2}$ and $a_{t}^{geo}$ is the geometry-based recovery action. The first branch is a forced near-goal stop. If the raw VLA predicts stop while the UAV is still far from the goal, the evaluator does not count the episode as successful; after the initial takeoff transient, such a far-stop prediction can terminate an episode unsuccessfully. This limitation is reported explicitly because the governor improves stopping near the goal but does not turn the raw VLA into a complete planner.

Table 3 lists the execution parameters used by this evaluator.

These thresholds were selected empirically during development in the AirSim Blocks environment to produce bounded motion, observable progress windows, and stable stopping under the stated primitive durations. They were not imported from another study, optimized on the final val-unseen split, or claimed as universal UAV-control constants. Their purpose is to make the present pilot evaluator explicit and reproducible; deployment in a different simulator or on hardware would require retuning against vehicle dynamics, sensor rate, localization uncertainty, and safety margins.

Before processing step *t*, the progress-aware governor stores the best distance from earlier observations:

(15) $$r_{t-1}^{best}=\min_{0\leq k<t}r_{k}.$$

The no-progress counter compares the current distance with this prior best value:

(16) $$n_{t}^{noprog}=\left\{\begin{array}{cc} 0, & \mathrm{if}\;r_{t}<r_{t-1}^{best}-0.08,\\ n_{t-1}^{noprog}+1, & \mathrm{otherwise}. \end{array}\right.$$

After this comparison, the stored best distance is updated as

(17) $$r_{t}^{best}=\min\left(r_{t-1}^{best},r_{t}\right).$$

This ordering matches the evaluator: improvement is tested against the best distance available before the current observation, so the reset condition is reachable. If the UAV does not improve for several consecutive steps, or if the current distance becomes substantially worse than $r_{t}^{best}$, the controller temporarily switches to the geometry recovery action:

(18) $${\tilde{a}}_{t}=\left\{\begin{array}{cc} a_{t}^{geo}, & \mathrm{if}\;n_{t}^{noprog}\geq \tau \;\mathrm{or}\;r_{t}>r_{t}^{best}+\delta ,\\ {\tilde{a}}_{t}^{basic}, & \mathrm{otherwise}. \end{array}\right.$$

This mechanism is intentionally transparent: it does not hide policy failure, but makes the failure mode measurable and correctable.

The geometry recovery primitive is selected by a fixed priority order rather than by another learned model:

(19) $$a_{t}^{geo}=\left\{\begin{array}{cc} 0, & r_{t}<1.1,\\ 7\;\mathrm{or}\;6, & d_{t}<1.2\;\mathrm{and}\;{∥\Delta p_{xy}∥}_{2}>2.0,\\ 4, & z^{goal}-z_{t}<-0.6,\\ 5, & z^{goal}-z_{t}>0.6,\\ 3, & \psi_{t}^{goal}-\psi_{t}>0.22,\\ 2, & \psi_{t}^{goal}-\psi_{t}<-0.22,\\ 1, & \mathrm{otherwise}. \end{array}\right.$$

The action labels correspond to stop, forward, yaw-right, yaw-left, ascend, descend, avoid-left, and avoid-right primitives. This priority order is intentionally simple: it first handles stopping, then front-depth avoidance, then altitude mismatch, then yaw alignment, and finally forward motion.

Finally, near-goal precision control scales the action speed and duration as the UAV approaches the target:

(20) $$\left(v_{t},\Delta t_{t}\right)=\left\{\begin{array}{cc} (\min(v,0.65),\min(\Delta t,0.08)), & r_{t}<1.8,\\ (\min(v,1.10),\min(\Delta t,0.09)), & 1.8\leq r_{t}<3.0,\\ (v,\Delta t), & r_{t}\geq 3.0. \end{array}\right.$$

This reduces overshoot near the stopping threshold and is critical for turning a high-quality approach trajectory into a successful episode.

For closed-loop diagnosis, the evaluator records not only success and final distance, but also the action-source statistics:

(21) $$\rho_{override}=\frac{N_{override}}{N_{steps}},\;\rho_{keep}=\frac{N_{keep}}{N_{steps}},\;\rho_{fallback}=\frac{N_{fallback}}{N_{steps}},$$

where $N_{override}$ counts executed actions changed by the governor, $N_{keep}$ counts steps where the executed action is identical to the raw VLA proposal, and $N_{fallback}$ counts steps whose action source is geometry recovery or forced near-goal stopping. These quantities are diagnostic events, not a mutually exclusive partition. In particular, geometry recovery can select the same discrete action as the raw VLA proposal, so a fallback-tagged step may still be counted as retained rather than overridden. We also report collision rate and SPL, computed from successful path length relative to the direct start-to-goal distance. These diagnostics are essential because a high success rate with a high fallback rate should be interpreted as a reliable hybrid system, not as a raw learned controller.

**Figure 3:**
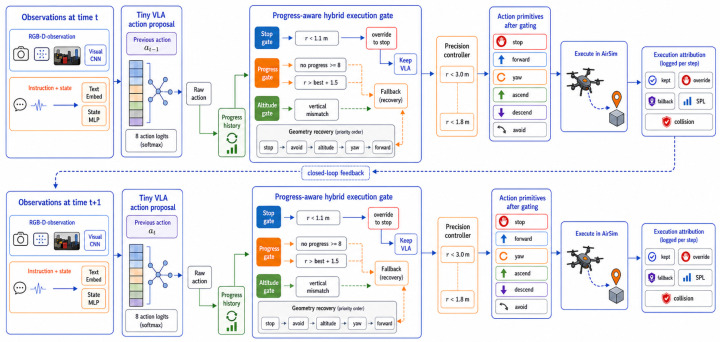
Progress-aware hybrid execution gate over two consecutive closed-loop steps. Blue denotes the normal VLA/action flow and stop/keep decisions, orange denotes progress and precision logic, green denotes progress history and altitude gating, and purple denotes action-history or descent elements. Rounded boxes represent modules or decision conditions; solid arrows show the main execution path, orange/green dashed arrows show recovery triggers, and the blue dashed arrow shows closed-loop feedback. The gate keeps safe VLA actions and replaces unsafe or non-progressing actions with geometry-based recovery before AirSim execution.

Figure 3 visualizes the progress-aware execution gate and its feedback path.

### 3.6. Traceability and Reproducibility

All major artifacts are stored under a single experiment directory. Processed datasets are placed under data/processed, training and evaluation outputs under runs, figures under runs/figures, and human-readable reports under runs/reports. Each experiment keeps its input data path, model checkpoint, metrics file, and summary report. The visual VLA models are trained for 8 epochs with batch size 96, image size 64×64, AdamW optimization, learning rate $2\times 10^{-3}$, weight decay $10^{-4}$, and cosine learning-rate scheduling; Adam is the relevant baseline optimizer family for this setup [47]. In the final offline protocol, val-seen accuracy alone selects the checkpoint; val-unseen metrics are not computed during training and are evaluated once after the selected checkpoint is reloaded. The main ablations use seed 11, and the full unweighted VLA is independently trained with seeds 11, 21, and 22. The final progress+precision configuration crosses these three locked checkpoints with target seeds 777, 778, and 779, using 20 episodes per combination and 180 episodes in total. The training script supports language_mode=no_coords and state_mode=no_goal_geometry for shortcut diagnostics, and the closed-loop evaluator records success, SPL, collision, path length, override rate, VLA-kept rate, fallback-tagged rate, and precision-control usage. This organization is part of the method rather than only an engineering convenience: because the main claim depends on distinguishing offline action accuracy from closed-loop success, the paper preserves traceability from dataset generation to model training and live AirSim evaluation.

## 4. Experiments

### 4.1. Experimental Setup

The experiments are designed to answer four questions. First, can a tiny RGB-D VLA policy learn multimodal action imitation from the AirSim benchmark? Second, does the offline result depend on explicit coordinate tokens or goal-geometry fields? Third, does high offline action accuracy transfer to closed-loop UAV navigation? Fourth, how much does the hybrid execution layer contribute when the learned policy is evaluated under a multi-seed closed-loop protocol?

The main pilot dataset is the AirSim RGB-D VLA Blocks dataset described in Section 3.2. It contains 100 expert episodes and 3385 step-level samples, split into 2312 training steps, 473 val-seen steps, and 600 val-unseen steps. The val-seen split uses target distributions similar to the training episodes, whereas the val-unseen split samples more distant and laterally shifted targets. This setting is intentionally modest in scale but useful for controlled ablation: every sample has synchronized RGB, depth, instruction, state, previous action, and expert action fields.

Closed-loop evaluation is conducted in the same AirSim Blocks environment. Here, an episode is one complete sampled-waypoint rollout: the UAV is reset and takes off, one target is sampled, and actions are executed until a valid near-goal stop, a far-stop prediction after the initial takeoff transient, or the 90-step limit. Collision is logged as a separate diagnostic event rather than used as an immediate termination condition. The execution-layer ablation uses the seed-11 checkpoint selected only on val-seen and target seeds 777, 778, and 779, with 20 episodes per target seed and 60 episodes per variant. A stricter robustness test then crosses all three independently trained, val-seen-selected checkpoints with the same three target seeds, again using 20 episodes per combination, for 180 progress+precision episodes. Every comparison uses the same target sampling function, success threshold, maximum step budget, and action primitive set. We report success count, success rate with Wilson 95% confidence interval, SPL, average final distance, override rate, VLA-kept rate, fallback-tagged rate, and collision rate. For offline imitation, val-seen is the model-selection split and val-unseen is evaluated once as the final held-out test. For efficiency, we report parameter count, checkpoint size, mean latency, P95 latency, and equivalent inference frequency.

The experiments were implemented in Python with PyTorch and executed in the Microsoft AirSim Blocks environment. Exact version identifiers were not retained in the archived experiment metadata; the official software pages are https://www.python.org/, https://pytorch.org/, and https://github.com/microsoft/AirSim (all accessed on 19 July 2026).

### 4.2. Dataset and Protocol

Table 4 summarizes the main AirSim RGB-D VLA dataset. The expert episode success rate is 0.73, which reflects the fact that the scripted expert is simple and bounded by a maximum episode length. This is important for interpretation: the dataset is not a perfect demonstration corpus or mature benchmark, but a traceable pilot dataset for studying lightweight VLA action imitation and governed closed-loop execution. The original split names are retained for traceability, but their final protocol roles are explicit: train fits the model, val-seen selects the epoch, and val-unseen is evaluated once after training as the final held-out test. Val-unseen is deliberately harder because its targets are farther and more laterally shifted; its action accuracy is therefore teacher-forced step-level generalization under a harder target distribution, not expert navigation success.

### 4.3. Offline Multimodal Ablation

**Figure 4:**
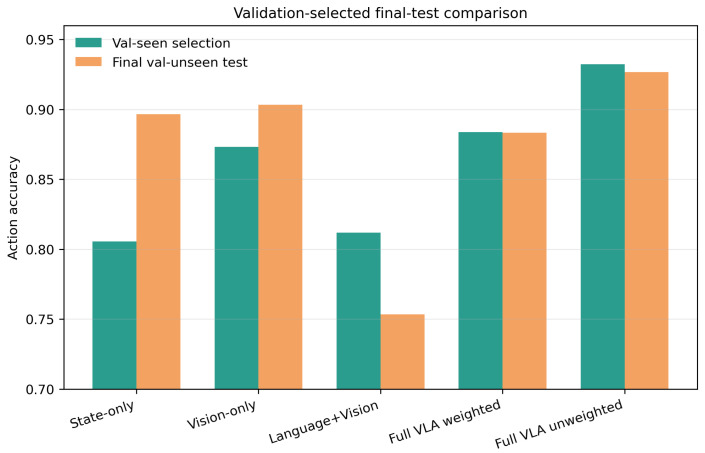
Validation-selected final-test comparison. Bars show val-seen checkpoint-selection accuracy and the single final val-unseen test evaluation for each original modality configuration.

**Figure 5:**
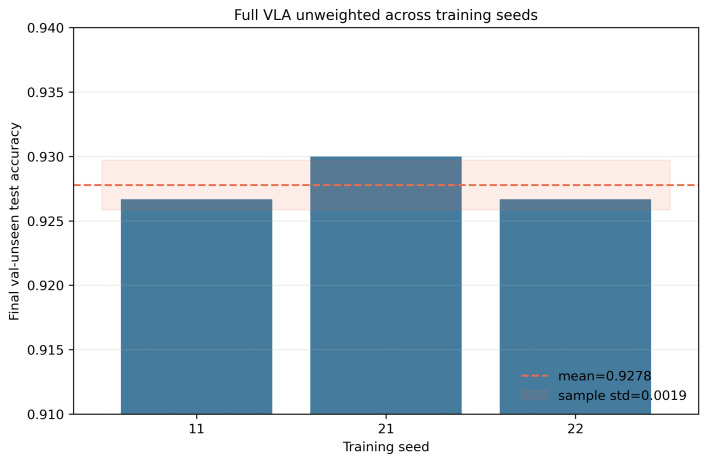
Final val-unseen test accuracy for three independently trained full unweighted RGB-D VLA checkpoints. Each checkpoint is selected using val-seen only; the dashed line and shaded band show the mean and sample standard deviation.

Table 5 summarizes the expert action-label distribution. Table 6 reports the validation-selected final-test results, and Figure 4 visualizes the same modality comparison. Vision-only and state-only reach 0.9033 and 0.8967 final val-unseen accuracy, respectively. Language+Vision alone is weaker at 0.7533, indicating that language and RGB-D observation without explicit state are insufficient for this local action-label distribution. The unweighted full VLA remains the strongest original configuration, reaching 0.9267 final-test accuracy after epoch selection using val-seen only. To measure training variability, we independently train this model with seeds 11, 21, and 22. Their final val-unseen scores are 0.9267, 0.9300, and 0.9267, giving 0.9278±0.0019 (mean ± sample standard deviation), as shown in Figure 5. The diagnostic shortcut ablations below remain single-seed unless explicitly noted.

**Table 5:** Expert action-label distribution in the AirSim RGB-D VLA Blocks dataset. Labels correspond to stop (0), forward motion (1), right/left yaw correction (2/3), ascent (4), descent (5), and lateral avoidance (6/7). The present Blocks trajectories mainly activate labels 0–4, so aggregate action accuracy is interpreted together with the confusion matrix in Figure 6.

Split	Stop	Forward	Yaw R	Yaw L	Ascend	Descend	Avoid L	Avoid R
Train	60	1703	116	114	319	0	0	0
Val-seen	13	338	16	28	78	0	0	0
Val-unseen	0	449	30	31	90	0	0	0

**Table 6:** Validation-selected offline multimodal comparison on the AirSim RGB-D VLA benchmark. Val-seen selects the checkpoint, and val-unseen is evaluated once after training as the final held-out test. Accuracy measures teacher-forced expert action classification, not closed-loop navigation success.

Model	Input	Selected Epoch	Val-Seen	Final Val-Unseen Test	Params	Ckpt. (MB)
State-only	state + previous action	6	0.8055	0.8967	95,976	0.380
Vision-only	RGB-D + previous action	1	0.8732	0.9033	102,120	0.404
Language+Vision	instruction + RGB-D + previous action	4	0.8118	0.7533	114,408	0.451
Full VLA weighted	instruction + RGB-D + state + previous action	1	0.8837	0.8833	132,840	0.521
Full VLA unweighted	instruction + RGB-D + state + previous action	8	0.9323	0.9267	132,840	0.521

**Figure 6:**
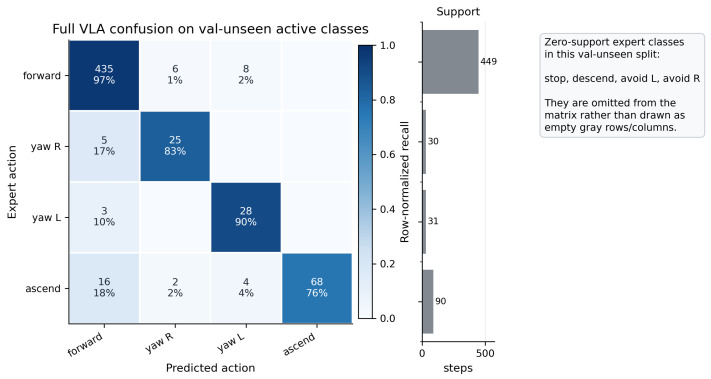
Row-normalized confusion matrix of the full unweighted VLA on the final held-out AirSim RGB-D VLA split, restricted to expert classes with nonzero support in val-unseen. Each non-empty cell reports both raw count and row percentage, the support panel shows the number of test samples per active expert action, and the note lists the zero-support classes omitted from the matrix. The figure is used to qualify the aggregate accuracy in Table 6: forward motion dominates the dataset, while yaw and ascent classes are inspected separately to avoid interpreting the score only through the majority class.

These results support the central offline claim: multimodal fusion is useful in this benchmark. However, the table and figures should not be read as evidence of successful navigation. They measure whether the model predicts expert action labels under teacher-forced held-out data, and the class distribution in Table 5 shows why aggregate accuracy must be paired with class-level diagnostics. This distinction is consistent with imitation-learning results showing that offline supervised policies can degrade under their own induced state distribution [28]. Closed-loop navigation is evaluated separately below.

The language-related results require a deliberately narrow interpretation. Language+Vision without explicit state reaches 0.7533, whereas the full model reaches 0.9267; this shows that template language and RGB-D alone are insufficient for immediate low-level action prediction in the present data distribution. The no-coordinate diagnostic preserves 0.9283 accuracy, so copied numeric waypoint tokens are not the main source of the score. However, we do not include a full-VLA-without-language intervention, and the instructions are templated rather than human-written. Therefore, the present experiments do not quantify an independent marginal contribution of language within the full model and do not establish open-ended language grounding. The controlled template-semantic controller reported later is likewise a rule-parser interface test, not evidence that the learned VLA has acquired open-vocabulary language competence.

### 4.4. Shortcut Diagnostics: Coordinate Language and Goal Geometry

**Figure 7:**
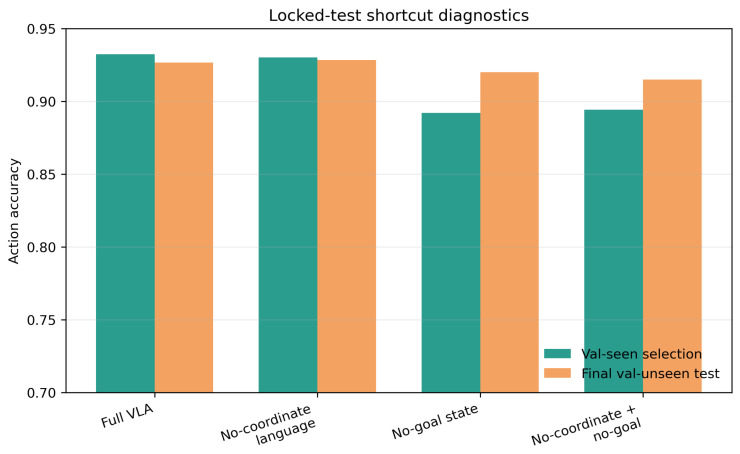
Validation-selected shortcut-diagnostic results. No-coordinate language preserves final-test accuracy, while removing explicit goal geometry causes a modest reduction.

**Figure 8:**
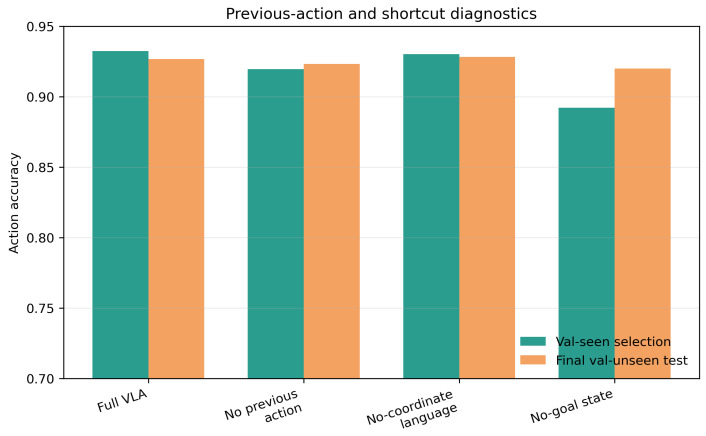
Previous-action and shortcut diagnostics under the final protocol. Removing the previous-action embedding changes final-test accuracy from 0.9267 to 0.9233, a smaller reduction than removing explicit goal geometry.

Table 7 and Figure 7 and Figure 8 report the shortcut-diagnostic ablations under the same validation-selected final-test protocol. Removing numeric coordinate tokens reduces the vocabulary from 116 to 15 and yields 0.9283 final val-unseen accuracy, close to the full model’s 0.9267. This indicates that templated coordinate numbers are not the main source of the offline score. Removing explicit goal geometry from the state reduces final-test accuracy to 0.9200. Removing the previous-action embedding gives 0.9233, while combining no-coordinate language with no-goal state gives 0.9150. We also audited the dataset schema for next-step leakage: the step record has no next_* field, and the state-vector displacement terms are goal-relative rather than next-state deltas.

**Figure 9:**
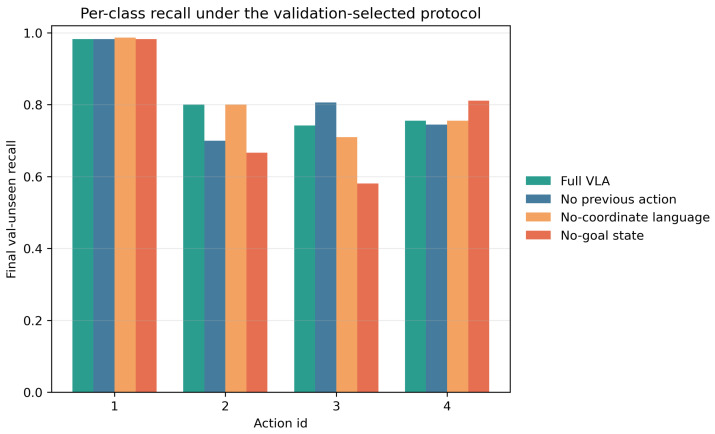
Per-class final val-unseen recall for shortcut diagnostics. Action id 1 is forward motion, ids 2 and 3 are yaw-correction primitives, and id 4 is vertical motion. Goal-geometry removal mainly reduces yaw-correction recall, while forward-motion recall remains high.

Figure 9 further breaks these shortcut diagnostics down by val-unseen action class. The strongest degradation from removing explicit goal geometry appears on the yaw-correction actions (ids 2 and 3), while the forward action remains high across variants. This class-level view is important because aggregate accuracy alone can hide which low-level control primitives are most affected by each removed input channel.

**Figure 10:**
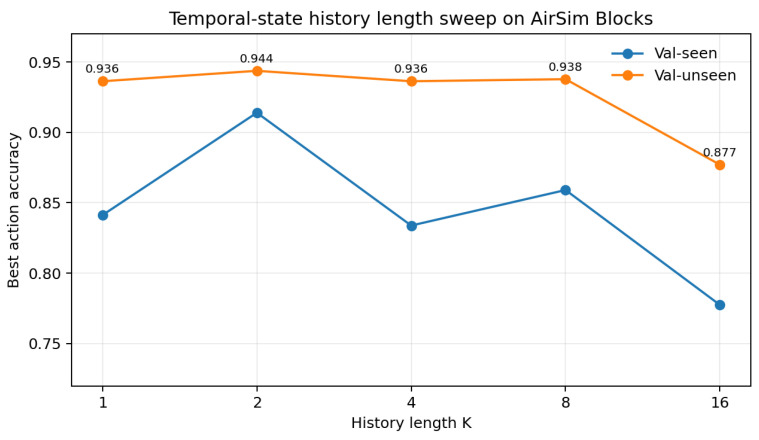
Auxiliary history-length sweep on the AirSim Blocks state-sequence action dataset. Short histories (K = 1–8) perform similarly, while K = 16 degrades, suggesting that the current low-level action labels do not benefit from arbitrarily long history in this small controlled setting. This figure is a temporal-context diagnostic, not a replacement for the RGB-D VLA ablations.

As an auxiliary temporal-state diagnostic, we also sweep the sequence history length on the AirSim Blocks expert action dataset using the state-sequence model rather than the RGB-D VLA network. Figure 10 shows that K = 1, 2, 4, and 8 achieve similar val-unseen action accuracy, with K = 2 reaching the best single-seed score of 0.9437, while K = 16 drops to 0.8770. This supports a conservative interpretation: short temporal context is useful for low-level UAV action prediction, but longer history is not automatically better under the current small-data GRU setting. Because this sweep uses a state-sequence diagnostic model, it is not treated as the main RGB-D VLA result.

### 4.5. Closed-Loop Evaluation

**Figure 11:**
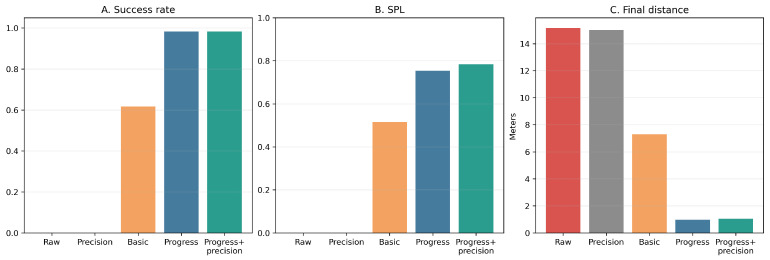
Locked-checkpoint closed-loop ablation across 60 episodes per variant. Raw VLA and precision-only VLA fail completely; progress-aware governance provides the largest reliability gain; near-goal precision improves SPL and removes the single collision recorded for the progress-only variant.

**Figure 12:**
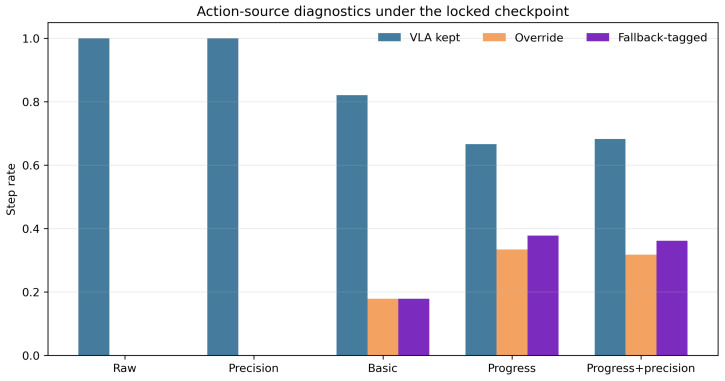
Action-source statistics for the locked-checkpoint variants. Progress+precision keeps 68.3% of raw VLA actions, overrides 31.7%, and uses fallback-tagged execution on 36.1% of steps. Kept and fallback rates are not mutually exclusive because recovery can select the same discrete action as the raw proposal.

Table 8, Figure 11 and Figure 12 report the final locked-checkpoint closed-loop comparison and action-source attribution. Raw VLA and precision-only VLA both obtain 0/60 success, showing that offline action accuracy and near-goal speed reduction alone do not yield reliable autonomous navigation. Basic governance improves success to 37/60 but remains unstable. Progress-aware governance and progress-aware governance with near-goal precision both reach 59/60 success. Precision control raises SPL from 0.753 to 0.784 and changes the collision count from one to zero, although it does not add another successful episode in this locked-checkpoint run.

The locked-checkpoint closed-loop results confirm that action imitation and navigation success are not interchangeable metrics. The progress+precision system achieves 59/60 success with zero recorded collisions in the Blocks environment, but its override and fallback rates remain substantial. This is the intended interpretation: the learned VLA becomes practical only when embedded in an explicit execution layer, and the paper evaluates that hybrid system transparently rather than attributing all success to raw VLA inference.

### 4.6. Checkpoint-by-Target Cross Evaluation

**Figure 13:**
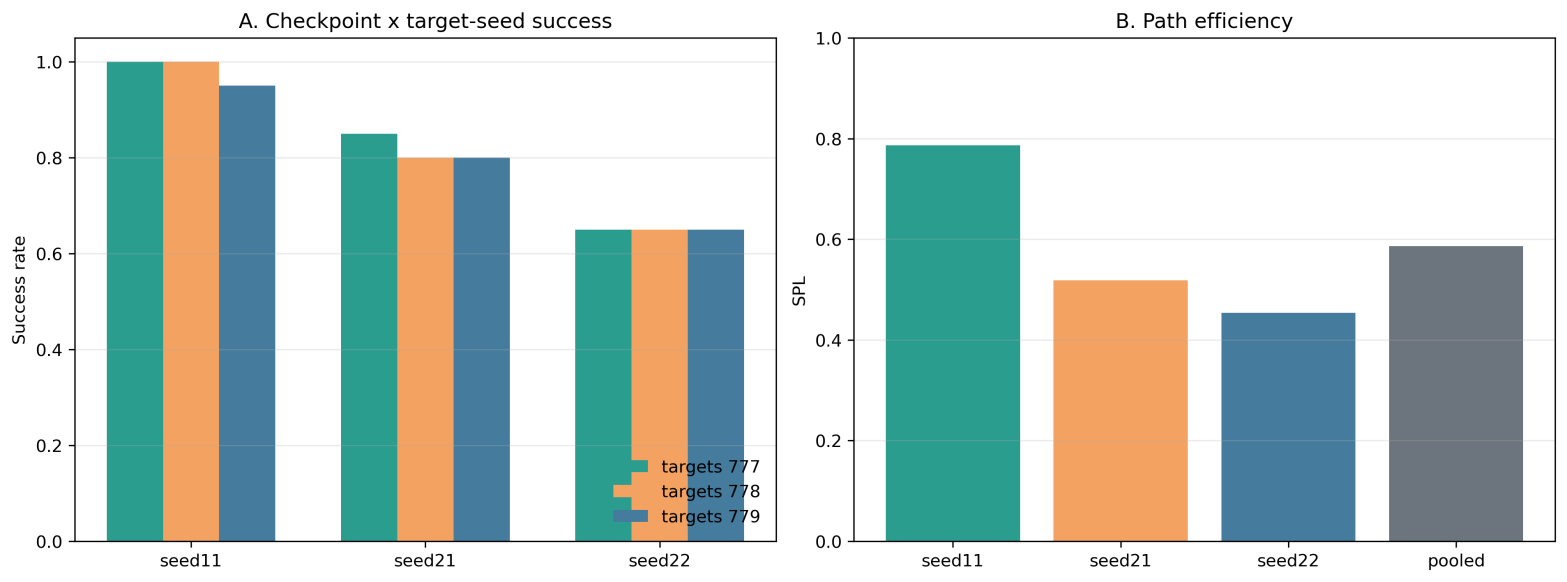
Closed-loop success and path efficiency in the three-checkpoint by three-target-seed crossed evaluation. Similar offline test accuracy does not imply similar closed-loop behavior; the crossed protocol exposes marked sensitivity to the independently trained checkpoint.

Table 9 and Figure 13 provide the stricter robustness check requested for the final protocol. Each independently trained checkpoint is selected using val-seen only and is then evaluated with progress+precision execution over the same 60 goals from target seeds 777/778/779. The checkpoints have nearly identical final val-unseen action accuracy, yet their closed-loop success differs substantially: seed 11 reaches 59/60, seed 21 reaches 49/60, and seed 22 reaches 39/60. Across all 180 episodes, the system succeeds in 147 episodes (81.7%, Wilson 95% CI: 75.4–86.6%) with no recorded collisions. This result is more conservative than reporting the strongest checkpoint alone and demonstrates that teacher-forced action accuracy does not capture closed-loop training-seed sensitivity. The seed-11 cell was executed independently from the locked execution-layer ablation: both runs obtain 59/60 success, while small differences in SPL and step-level attribution are retained rather than averaged, reflecting trajectory-level variability in repeated live AirSim runs.

**Figure 14:**
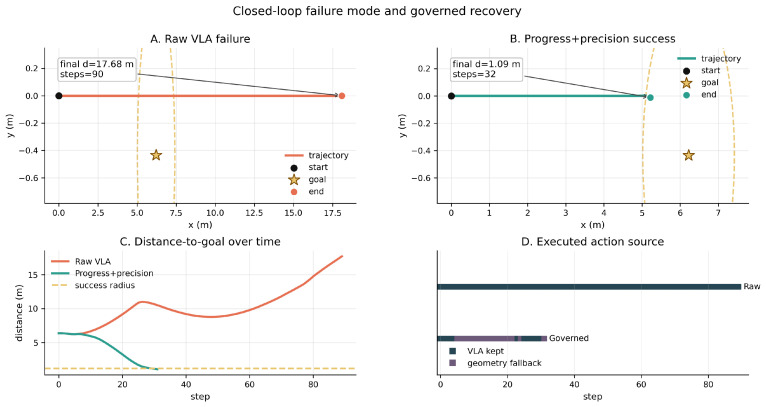
Representative closed-loop failure and recovery analysis. Panels (**A**,**B**) show the raw VLA failure and progress+precision success trajectories, including the target neighborhood; the yellow dashed circles mark the 1.2 m success radius. Panel (**C**) compares distance-to-goal over time, and Panel (**D**) visualizes the executed action source at each step using only the action-source categories present in these trajectories. The qualitative comparison illustrates the same deployment gap measured quantitatively in Table 8: raw VLA drifts away, while the governed variant reaches the goal through a mixture of retained VLA actions and execution-layer recovery.

Figure 14 illustrates a representative raw-policy failure and governed recovery after Figure 13.

### 4.7. Diagnostic Baselines and Attribution Boundary

**Figure 15:**
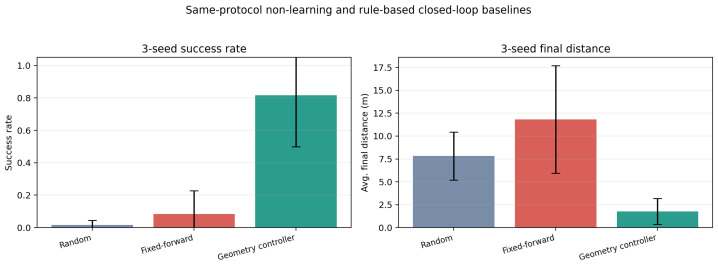
Three-seed closed-loop baseline expansion for non-learning and rule-based policies. The random and fixed-forward baselines remain weak overall, whereas the geometry controller is strong but exhibits seed sensitivity. This prevents overclaiming an oracle-like geometry upper bound and keeps the paper’s attribution focused on the learned VLA plus hybrid execution stack.

The controlled waypoint task may still be partly solvable by hand-designed geometry. Table 10 and Figure 15 therefore report a three-seed expansion of non-learning and rule-based baselines under the same AirSim Blocks goal-sampling protocol. Random and fixed-forward policies remain weak overall, so the task is not solved by arbitrary motion or a single forward primitive. The hand-designed geometry controller is much stronger, reaching 49/60 success overall, but it is not a stable 100% upper bound because seed 778 produces only 9/20 successes. The learned VLA variants should therefore be interpreted as studying whether multimodal action imitation can be made useful inside a hybrid execution stack, not as outperforming a hand-designed controller in this simplified environment.

### 4.8. Efficiency Analysis

Table 11 summarizes the full visual VLA latency profile. The model has 132,840 parameters and a 0.521 MB checkpoint. In batch-one inference, the CPU mean latency is 0.9347 ms, faster than the CUDA mean latency of 2.7566 ms. This inversion is expected for such a small model, where GPU kernel-launch overhead dominates the actual computation.

This result supports the efficiency claim for action-level VLA inference. It does not imply that a large token-level VLA or a full real-world UAV stack would run at the same speed. The profiling covers the tiny RGB-D visual encoder, text embedding, state encoder, previous-action embedding, and action head used in this paper.

### 4.9. Preliminary AerialVLN-S Action Prediction

**Figure 16:**
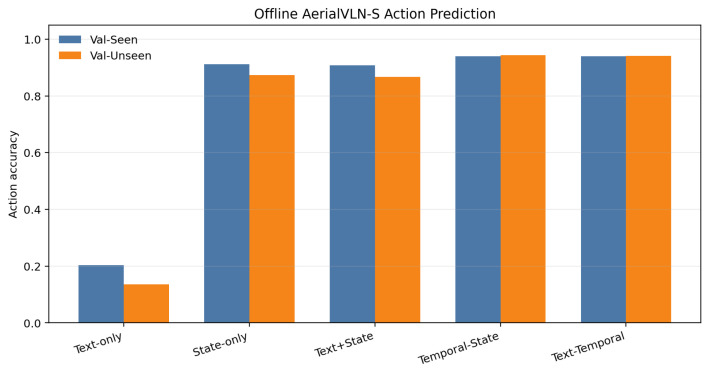
Preliminary AerialVLN-S action-prediction visualization. Bars compare majority, text-only, state-only, text+state, and temporal variants on val-seen and val-unseen splits. The figure supports the hierarchical interpretation used in this paper: language alone is weak for immediate low-level action prediction, while temporal state history captures much of the local control structure.

Before constructing the AirSim RGB-D VLA benchmark, we evaluated lightweight action predictors on AerialVLN-S trajectory annotations. This preliminary experiment is not the main VLA result because the visual observation is not part of the model input. It is nevertheless useful for understanding the structure of low-level UAV action labels. Table 12 shows that text-only prediction is weak, while temporal state history is highly predictive; Figure 16 visualizes the same comparison.

The result suggests that low-level step-wise actions are strongly governed by local geometry and temporal history. This supports the hierarchical interpretation used throughout the paper: language and vision should help define goals, semantic context, and local observations, while the execution layer must still manage progress, stability, and stopping.

### 4.10. Auxiliary AerialVLN-S Perturbation Proxy

**Figure 17:**
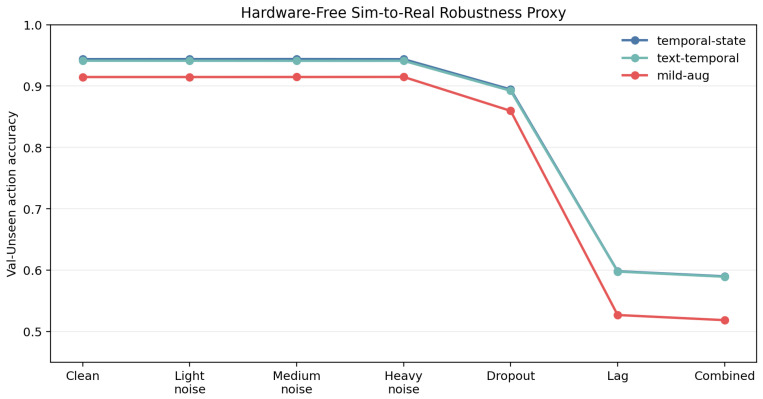
Auxiliary AerialVLN-S perturbation-proxy curves. Dynamics lag causes the largest degradation among the tested perturbations, whereas naive augmentation does not fully recover delay robustness and can reduce clean accuracy. The plot is not a robustness result for the full VLA system; it only motivates future delay-aware state estimation and execution.

We include a hardware-free perturbation proxy on AerialVLN-S temporal action models only as an auxiliary diagnostic. It is not an evaluation of the full RGB-D VLA-plus-execution system, is not performed in AirSim Blocks, and is not used to support any real-UAV, sim-to-real, or visual-transfer robustness claim. Within this separate diagnostic, Table 13 shows that moderate state noise has little effect, whereas sensor dropout and dynamics lag cause substantial degradation. Dynamics lag reduces Temporal-State accuracy from 0.9439 to 0.5984, and the combined perturbation reduces it further to 0.5900; Figure 17 visualizes these perturbation curves.

This auxiliary diagnostic should be interpreted carefully. It neither demonstrates the robustness of the proposed full VLA system nor changes the scope of the closed-loop findings, which remain restricted to the controlled AirSim Blocks benchmark. Its value is limited to showing that naive augmentation does not solve delay-related failures in these separate temporal models, motivating future delay-aware state estimation and execution. 

### 4.11. Closed-Loop Visual Cue Ablation

**Figure 18:**
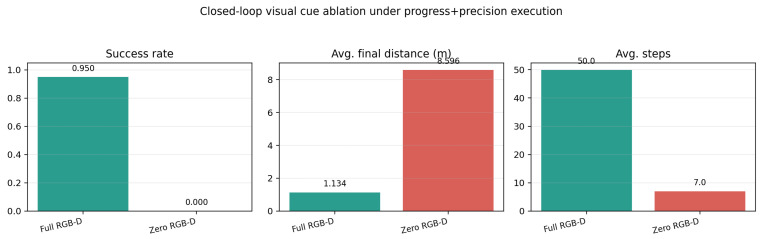
Closed-loop visual-cue ablation with the progress+precision execution stack. Removing RGB-D tensors at inference time collapses seed-777 success from 19/20 to 0/20, indicating that the current policy stack depends on valid visual inputs. The evidence is intentionally bounded to this controlled setting and should not be read as a general semantic grounding benchmark.

To test whether the closed-loop system is merely following state and governor logic while ignoring visual tensors, we add a minimal visual-cue ablation under the same seed-777 progress+precision execution setting. The full RGB-D setting reuses the existing 20-episode progress+precision run. The zero-RGBD setting keeps the checkpoint, instruction, state vector, previous-action input, governor, and precision control unchanged, but replaces the RGB and depth tensors by zeros at inference time. Table 14 and Figure 18 show a sharp degradation: the full RGB-D run succeeds in 19/20 episodes, while zero-RGBD succeeds in 0/20 and stops after about seven steps on average. This supports the minimal claim that valid visual tensors are necessary for the current closed-loop VLA stack. It should not be overinterpreted as open-world semantic visual grounding, because the task still uses templated waypoint language and a controlled Blocks environment.

### 4.12. Template-Semantic Waypoint Controller Pilot

The no-coordinate language ablation in Section 4.4 shows that numeric coordinate tokens are not necessary for high offline action accuracy, but it does not by itself test whether a language-like waypoint interface can be executed in closed loop. We therefore add a small controlled pilot that removes explicit x/y/altitude values from the command and uses only discrete sector words: near/mid/far for forward range, left/center/right for lateral sector, and low/middle/high for altitude band. A rule parser maps these template-semantic tokens to a canonical waypoint sector, and the same geometry action primitives used in the closed-loop baselines execute the resulting waypoint. This is intentionally a narrow interface test: it evaluates a template-semantic waypoint-to-controller pipeline, not open-ended natural-language grounding or object-centric visual grounding.

**Figure 19:**
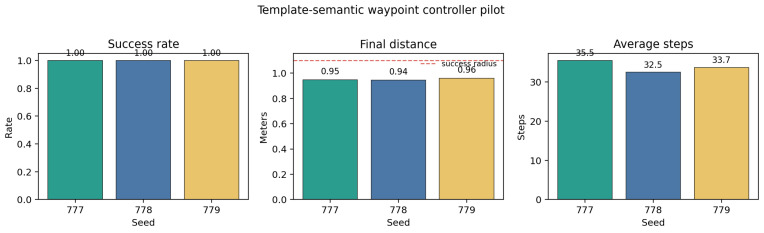
Three-seed template-semantic waypoint controller pilot. The result shows that discrete, coordinate-free sector commands can be parsed into waypoint sectors and executed reliably in the controlled Blocks environment. It should not be read as evidence of open-world semantic language or visual grounding.

Table 15 and Figure 19 summarize the three-seed AirSim Blocks run. The parser succeeds on all generated template commands, and the controller reaches 60/60 goals with an average final distance of 0.950±0.008 m. The result confirms that the current benchmark can support a coordinate-free, sector-level command interface, but it also reinforces the paper’s boundary: reliable execution here is provided by an explicit controller after rule parsing, not by a learned open-vocabulary semantic policy.

### 4.13. Representative Experimental Observations

**Figure 20:**
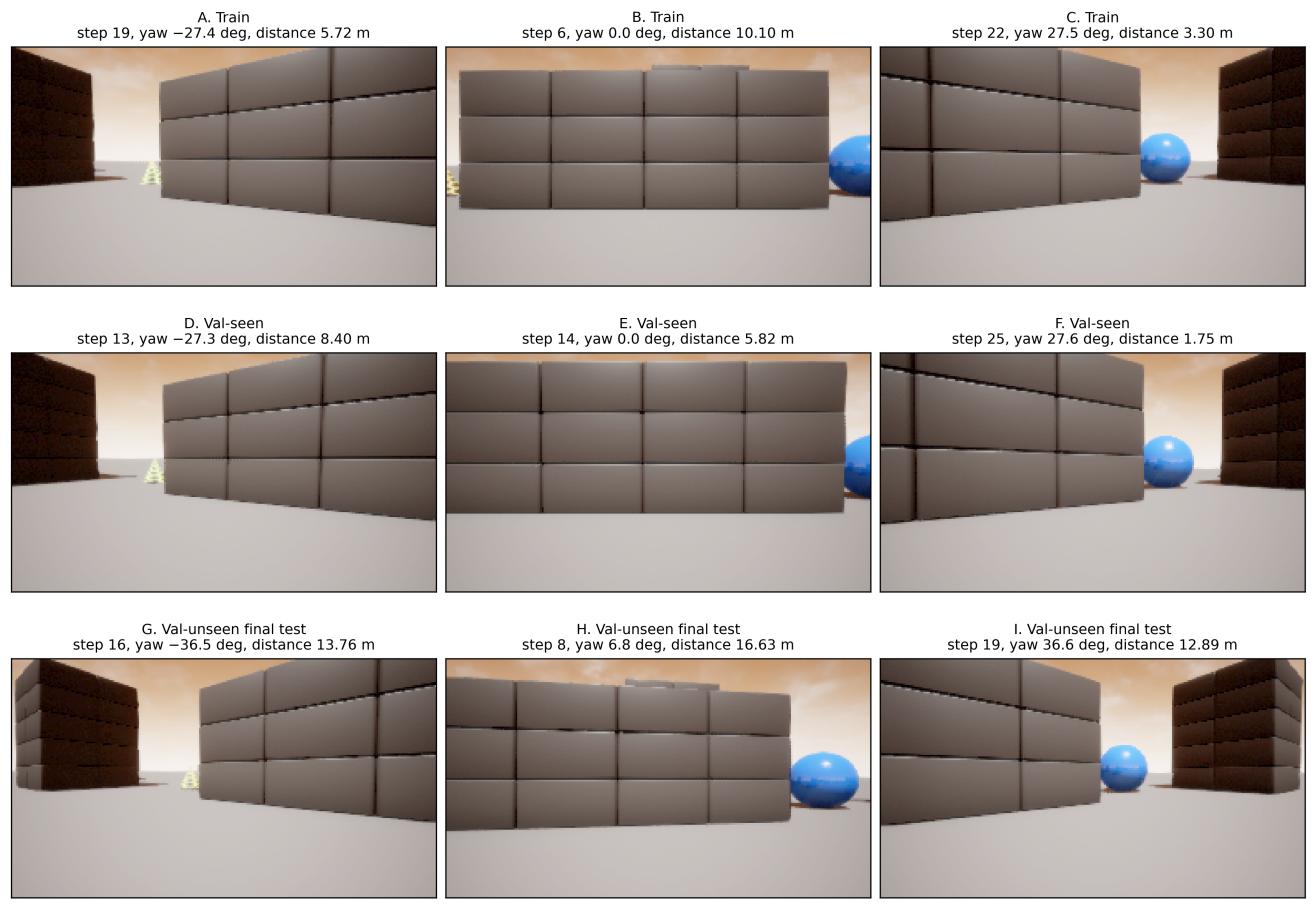
Representative unmodified AirSim RGB observations from train, val-seen, and final val-unseen splits at left-facing, near-centered, and right-facing yaw values. Panel labels report the recorded step, yaw, and target distance.

Figure 20 replaces the earlier wall-facing sequence with nine unmodified front-camera RGB observations sampled from the train, val-seen, and final val-unseen splits. Within each split, frames are selected from the lower, central, and upper portions of the observed yaw distribution, exposing left-facing, near-centered, and right-facing views together with the recorded step, yaw, and target distance. These are actual AirSim frames rather than generated illustrations. They make the simulator setting inspectable while also showing its limitation: the same Blocks assets recur across splits, so the figure documents observation provenance and viewpoint variation rather than broad visual diversity or semantic grounding.

## 5. Discussion and Threats to Validity

The strongest part of the study is the consistency between offline diagnosis and closed-loop failure analysis. The model learns the controlled action labels well, the no-coordinate ablation reduces the risk that the score is driven only by copied numeric language tokens, and the raw closed-loop policy still fails. This pattern supports the paper’s central claim that offline VLA action accuracy is useful but insufficient. The success of progress-aware hybrid execution is therefore an experimentally measured system property rather than a rhetorical claim.

The weakest point is also clear: the current AirSim Blocks waypoint task is geometrically simple. The three-seed geometry controller in Table 10 is much stronger than random and fixed-forward baselines, which means this benchmark cannot establish superiority over classical geometric control. Instead, it establishes a controlled testbed for measuring when a compact multimodal policy contributes to an execution stack and how much of the final behavior is supplied by safety logic. For this reason, the paper reports override and fallback rates alongside success rate. Without those attribution metrics and the checkpoint crossing in Table 9, the strongest-checkpoint result of 59/60 would be easy to overinterpret. The pooled 147/180 result and the spread from 39/60 to 59/60 show that training-seed sensitivity remains an important limitation even when offline test accuracies are similar.

The visual evidence has a similar boundary. The onboard frames in Figure 20 are useful for confirming that the AirSim rollout was recorded, but the Blocks scene provides limited visual diversity and the forward-facing wall texture changes only subtly. Therefore, the qualitative figure is paired with trajectory, distance, and depth curves rather than being treated as standalone evidence of semantic visual grounding. A stronger future version should include object-rich AirSim environments, viewpoint changes with clearer visual landmarks, and failure cases where perception rather than geometry determines the correct action.

The execution module also relies on privileged simulator geometry: current position, target position, target-relative distance, altitude error, and front-depth statistics. In particular, the three target coordinates, three target-relative displacement coordinates, and scalar target distance are supplied directly by the simulator. The no-goal-state diagnostic lowers final-test accuracy from 0.9267 to 0.9200, so these fields help the current controlled action-prediction task; it is a single-checkpoint diagnostic that retains the remaining state, RGB-D, language, and action-history inputs, and therefore does not establish that geometric information alone dominates the result. A real platform would need a sufficiently reliable localization and goal-estimation stack; for example, RTK-GNSS/GNSS plus IMU fusion where satellite positioning is available, or visual-inertial odometry and SLAM where it is degraded, together with calibrated depth sensing. Position or target-estimation error would corrupt the progress signal and could cause premature stopping, delayed recovery, or recovery in the wrong direction. Depth noise could trigger unnecessary avoidance or miss an obstacle. Varying illumination, dynamic obstacles, wind disturbance, communication delay, battery limits, and onboard compute contention are likewise absent from Blocks. Consequently, the reported collision-free simulator rollouts are not evidence of real-world safety, and the empirical thresholds in Table 3 would need uncertainty-aware retuning and hardware validation.

Future physical-UAV deployment would also require an explicit systems interface that is outside the present experiment. RGB-D, IMU, and localization streams would need temporal synchronization and extrinsic calibration; the lightweight policy could run on a companion computer and provide bounded action primitives; and a flight-control platform would need to convert those primitives into stabilized low-level commands, enforce command limits, and provide failsafe, geofence, manual-override, and communication-loss handling [32,44]. This study implements none of these real-hardware components or flight tests. The paragraph specifies future deployment requirements, not a demonstrated deployment pathway.

Finally, the policy and governor are local rather than global. They predict or select the next primitive and maintain only short progress history; they do not build a map, search over routes, optimize a trajectory, represent the environment globally, or remember long-horizon decisions. The system therefore cannot be expected to plan detours around large obstacle configurations or resolve route-level ambiguity. More complex deployment would require a global planner or learned high-level waypoint generator above the present local VLA and execution interface, together with explicit temporal memory and replanning.

Several additional experiments would strengthen a journal submission. First, the current template-semantic waypoint pilot should be replaced by human-written or paraphrased instructions, so that language robustness can be separated from template parsing. Second, obstacle-rich or visually diverse environments should be added to activate descent and lateral avoidance labels that are sparse in the current dataset. Third, temporal visual memory should be tested against the current single-step visual encoder because UAV drift and delayed observations are inherently sequential. Fourth, object-centric goals should be introduced so that perception, rather than explicit waypoint geometry or sector parsing, determines the target. These experiments are not claimed in the present paper; they define the next stage needed to move beyond the current controlled pilot study.

## 6. Conclusions

This paper presented a lightweight and fully hardware-free pilot study of RGB-D vision-language-action modeling for UAV waypoint navigation in AirSim. We constructed a traceable AirSim RGB-D VLA Blocks dataset in which each step contains visual observation, depth, templated instruction, UAV state, previous action, and expert action supervision. On this dataset, a tiny full VLA policy with only 132,840 parameters achieved the strongest offline val-unseen action accuracy among the original modality ablations, demonstrating that RGB-D perception, language, state, and action history can be fused effectively even under a compact action-level architecture.

The most important finding, however, is not the offline accuracy itself. The shortcut-diagnostic ablations show that numeric coordinate tokens in the instruction are not necessary for high offline accuracy, but explicit goal geometry in the state remains useful. This defines the contribution boundary: the paper does not demonstrate strong open-ended language grounding, but it does show that a lightweight RGB-D VLA can learn action mappings in a controlled waypoint setting and that geometric shortcut effects can be measured rather than hidden.

Closed-loop evaluation provides the strongest evidence. In the locked seed-11 execution ablation, raw VLA and precision-only VLA fail despite strong offline action prediction, whereas progress-aware hybrid execution with near-goal precision reaches 59/60 goals with zero recorded collisions. The stricter checkpoint-by-target crossing reduces the pooled result to 147/180 and exposes a 39/60–59/60 range across independently trained checkpoints, despite their similar final offline accuracy. The experiments therefore support a cautious but useful conclusion: lightweight UAV-VLA should be viewed as a component inside a governed navigation stack, not as an unconstrained raw end-to-end controller. The contribution of the learned VLA is multimodal action imitation and compact inference; the governor supplies explicit progress recovery and near-goal control. Treating these components separately makes the system easier to diagnose and avoids overstating either the learned model’s autonomy or the governed system’s seed robustness.

Several limitations remain. The AirSim Blocks dataset is small and visually simple compared with real UAV navigation environments. The language instructions are templated rather than open-ended human commands, and the current task is a controlled waypoint task rather than navigation in complex unknown environments. The recorded expert action distribution is also incomplete: descent and lateral avoidance actions are part of the interface but are not sufficiently represented in the current Blocks trajectories, so the present evidence mainly supports waypoint approach, yaw correction, altitude ascent, stopping, and governed execution rather than full obstacle-rich UAV navigation. The visual encoder is intentionally tiny and does not provide large-scale semantic grounding. All demonstrated closed-loop reliability, including the collision logs and recovery behavior, is restricted to the controlled AirSim Blocks benchmark. The separate AerialVLN-S perturbation proxy is not a full-system, visual-transfer, sim-to-real, or real-UAV robustness result, and the current experiments do not include real drone deployment. Although the revised protocol now uses val-seen-only checkpoint selection, a one-time final val-unseen test, and a complete three-checkpoint by three-target-seed crossing for the strongest execution configuration, three training seeds and one simulator environment remain too limited for broad robustness claims; most auxiliary ablations also remain single-checkpoint studies. These limitations do not invalidate the main finding, but they define its scope: the paper establishes a low-cost, reproducible pilot for action-level RGB-D VLA with progress-aware hybrid execution, not a complete field-ready aerial VLA system.

Future work should expand the AirSim benchmark to multiple environments, richer object-centric goals, and more diverse language instructions. The policy should be extended with temporal visual memory, delay-aware state estimation, and stronger perception modules while preserving the lightweight execution interface. The safety/action governor should also be studied with finer ablations, including learned risk prediction and more challenging obstacle configurations. Finally, the hardware-free findings should be tested in higher-fidelity simulators and, when safety permits, on real UAV platforms. The long-term goal is a hierarchical UAV-VLA system in which language and vision specify intent, lightweight policies propose action primitives, and verifiable execution modules ensure safe progress in a closed loop.

## Figures and Tables

**Table 1:** Positioning of this work relative to related research families. The comparison is qualitative because the cited systems use different environments, action spaces, and evaluation protocols.

Research Family	Typical Setting	Policy or Perception Focus	Execution or Safety Layer	Position of This Work
UAV-VLN benchmarks [2,14,15,16]	Aerial instruction following	Route-level language navigation	Usually evaluated through navigation metrics	Provides context for aerial language navigation, but our task is a controlled AirSim Blocks waypoint pilot.
Large robotic VLA systems [3,4,19]	Multi-task robot control	High-capacity multimodal policies	Often relies on platform-specific action interfaces	Uses a much smaller action-level policy to study reproducible UAV-specific failure modes.
Imitation-learning navigation [28]	Expert-label action prediction	Offline supervised or aggregated imitation	Closed-loop errors can accumulate under induced states	Separates teacher-forced action accuracy from closed-loop UAV success.
Safety and local control [29,30,32]	Safety-critical robot execution	Constraint satisfaction and executable motion	Explicit monitoring, fallback, and control constraints	Embeds VLA action proposals in transparent progress-aware hybrid execution.
Lightweight simulation studies [5,33]	Hardware-free controlled evaluation	Low-cost reproducible experiments	Simulator-specific execution and logging	Provides a traceable AirSim RGB-D UAV-VLA pilot with attribution metrics.

**Table 2:** Implemented configuration of the full lightweight RGB-D VLA policy. The vocabulary size is reported for the full-language training configuration; the no-coordinate diagnostic uses a 15-token vocabulary.

Component	Configuration
Input	RGB-D tensor with 4 channels, resized to 64×64; full state vector of 16 dimensions; previous discrete action; and tokenized instruction.
Visual encoder	Conv 4→24 (kernel 5, stride 2, padding 2), Conv 24→48 (kernel 3, stride 2, padding 1), and Conv 48→72 (kernel 3, stride 2, padding 1); each convolution is followed by batch normalization and ReLU; adaptive average pooling to 1×1; Linear 72→128 and ReLU.
Text encoder	Lowercase regex tokenization using [a-z0-9.-]+; training-set vocabulary of 116 tokens in the full-language configuration, with PAD index 0 and UNK index 1; 64-dimensional learned embedding; masked mean pooling over non-padding tokens; maximum instruction length 64.
State and action encoders	Training-set normalized state; Linear 16→96, LayerNorm, ReLU, Linear 96→96, and ReLU. Previous-action embedding has 9 indices (8 actions plus padding) and dimension 16.
Fusion and prediction	Concatenated full-model feature dimension 128+64+96+16=304; Linear 304→192, LayerNorm, ReLU, Dropout(0.1), and Linear 192→8.
Training and size	AdamW, learning rate $2\times 10^{-3}$, weight decay $10^{-4}$, cosine schedule, 8 epochs, batch size 96; 132,840 parameters and 0.521 MB checkpoint.

**Table 3:** Main closed-loop execution parameters used by the AirSim evaluator. The values are taken from the evaluation script and are listed to make the hybrid execution layer reproducible.

Quantity	Value	Role in Execution
Base action speed *v*	2.2 m/s	Default translational primitive magnitude.
Base action duration Δt	0.10 s	Default primitive duration.
Yaw-rate primitive	±55°/s	Actions 2 and 3 rotate right/left by yaw rate.
Forced stop radius $\epsilon_{succ}$	1.1 m	Governor returns stop when $r_{t}<\epsilon_{succ}$.
Episode success check	stop with $r_{t}<1.2$ m	Evaluator counts success only when a stop occurs near the goal.
Progress improvement margin	0.08 m	Distance must improve by more than this value to reset the no-progress counter.
No-progress threshold τ	8 steps	Geometry recovery is triggered when $n_{t}^{noprog}\geq \tau$.
Regression threshold δ	1.5 m	Geometry recovery is triggered when $r_{t}>r_{t-1}^{best}+\delta$.
Front-depth warning	$d_{t}<1.2$ m and planar distance >2.0 m	Geometry recovery selects lateral avoidance.
Vertical guard margin	0.25 m	Blocks ascent/descent actions inconsistent with goal altitude error.
Vertical recovery threshold	$\left|z^{goal}-z_{t}\right|\;>0.6$ m	Geometry recovery selects ascend or descend.
Yaw recovery threshold	$\left|\psi_{t}^{goal}-\psi_{t}\right|\;>0.22$ rad	Geometry recovery selects yaw correction.
Precision control	$r_{t}<3.0$ m and $r_{t}<1.8$ m	Caps speed/duration to (1.10,0.09) and (0.65,0.08).
Maximum episode length	90 steps	Closed-loop rollout budget for the 60-episode-per-variant execution ablation.

**Table 4:** AirSim RGB-D VLA Blocks dataset summary. Each step contains synchronized RGB, depth, instruction, UAV state, previous action, and expert action fields. The split-level episode success rates expose the bounded nature of the scripted expert: val-unseen goals are intentionally harder and are used for step-level action generalization rather than as successful expert demonstrations.

Split	Episodes	Steps	Expert SR	Avg. Final Distance (m)
Train	70	2312	0.8571	0.9836
Val-seen	15	473	0.8667	1.0648
Val-unseen	15	600	0.0000	5.2603
Total	100	3385	0.7300	–

**Table 7:** Validation-selected shortcut diagnostics. No-coordinate language removes numeric x/y/altitude tokens; no-goal state removes goal position, goal-relative displacement, and scalar distance; no previous action removes the learned action-history embedding. Val-unseen is evaluated once after val-seen checkpoint selection.

Model Setting	State/Language Mode	State Dim	Vocab	Selected Epoch	Val-Seen	Final Test
Full VLA	full state/full language	16	116	8	0.9323	0.9267
No-coordinate language	full state/no coords	16	15	5	0.9302	0.9283
No-goal state	no goal geometry/full language	9	116	7	0.8922	0.9200
No previous action	full state/full language	16	116	5	0.9197	0.9233
No-goal state + no-coordinate language	no goal geometry/no coords	9	15	7	0.8943	0.9150

**Table 8:** Locked-checkpoint AirSim closed-loop ablation. Each variant uses the val-seen-selected seed-11 checkpoint and target seeds 777/778/779, with 20 episodes per target seed and 60 episodes total. Wilson CI denotes the Wilson 95% confidence interval for episode success rate.

Variant	Episodes	Success	SR	Wilson 95% CI	SPL	Final Dist. (m)	Override	VLA Kept	Fallback	Collision
Raw VLA	60	0	0.000	[0.000, 0.060]	0.000	15.161	0.000	1.000	0.000	0.000
Precision only	60	0	0.000	[0.000, 0.060]	0.000	15.008	0.000	1.000	0.000	0.000
Basic governor	60	37	0.617	[0.490, 0.729]	0.516	7.289	0.179	0.821	0.179	0.000
Progress governor	60	59	0.983	[0.911, 0.997]	0.753	0.967	0.333	0.667	0.378	0.017
Progress+precision	60	59	0.983	[0.911, 0.997]	0.784	1.048	0.317	0.683	0.361	0.000

**Table 9:** Three-checkpoint by three-target-seed crossed evaluation of the progress+precision configuration. Each checkpoint row aggregates 60 episodes; the pooled row aggregates all 180 episodes. Test accuracy is the one-time final val-unseen action accuracy after val-seen checkpoint selection.

Checkpoint	Test Acc.	Episodes	Success	SR	Wilson 95% CI	SPL	Override	Fallback	Collision
Seed 11	0.9267	60	59	0.983	[0.911, 0.997]	0.786	0.289	0.338	0.000
Seed 21	0.9300	60	49	0.817	[0.701, 0.894]	0.519	0.213	0.309	0.000
Seed 22	0.9267	60	39	0.650	[0.524, 0.758]	0.454	0.191	0.259	0.000
Pooled	–	180	147	0.817	[0.754, 0.866]	0.586	0.228	0.300	0.000

**Table 10:** Three-seed non-learning and rule-based baseline expansion under the same AirSim Blocks goal-sampling protocol. Seed 777 reuses the earlier diagnostic baseline, while seeds 778 and 779 are newly run in this revision. The result strengthens the attribution boundary: random and fixed-forward policies remain weak, while the geometry controller is strong but seed-sensitive rather than a stable 100% upper bound.

Method	Episodes	Success	SR Mean ± Std	Final Dist. Mean ± Std (m)	Steps Mean ± Std
Random	60	1	0.017±0.029	7.819±2.613	89.43±0.98
Fixed-forward	60	5	0.083±0.144	11.830±5.878	87.02±5.17
Geometry controller	60	49	0.817±0.318	1.765±1.413	44.83±19.31

**Table 11:** Full visual VLA batch-one inference latency. Profiling covers the tiny RGB-D visual encoder, text embedding, state encoder, previous-action embedding, and action head. Because this is an action-level network with only 132,840 parameters, CPU inference is faster than CUDA batch-one inference due to GPU launch overhead.

Device	Parameters	Checkpoint (MB)	Mean (ms)	P95 (ms)	Hz
CUDA	132,840	0.521	2.7566	4.2224	362.77
CPU	132,840	0.521	0.9347	1.2254	1069.92

**Table 12:** Preliminary AerialVLN-S action-prediction results. These models do not use visual input and are not the main AirSim RGB-D VLA result. They are included as diagnostic evidence that low-level UAV action labels are strongly driven by state and temporal history, while text-only action prediction is weak.

Model	Val-Seen Accuracy	Val-Unseen Accuracy
Majority action	0.4179	0.4539
Text-only	0.2036	0.1358
State-only	0.9122	0.8728
Text+State	0.9085	0.8672
Temporal-State GRU	0.9398	0.9439
Text-Temporal GRU	0.9401	0.9413

**Table 13:** Auxiliary perturbation proxy on AerialVLN-S temporal action models. Values are action-prediction accuracy under clean, dropout, dynamics-lag, and combined perturbation settings. This separate diagnostic does not evaluate the full RGB-D VLA-plus-execution system and is not real-UAV, AirSim-to-real, or visual-domain-transfer validation.

Model	Clean	Dropout	Dynamics Lag	Combined
Temporal-State	0.9439	0.8945	0.5984	0.5900
Text-Temporal	0.9413	0.8923	0.5978	0.5893
Strong Aug.	0.7490	0.7182	0.5397	0.5372
Mild Aug.	0.9147	0.8596	0.5270	0.5186

**Table 14:** Single-seed closed-loop visual-cue ablation under progress+precision execution. Zero-RGBD keeps the same checkpoint and non-visual inputs but zeros RGB and depth tensors at inference time. The result supports a minimal visual-dependence claim for the current system, not a broad semantic-grounding claim.

Setting	Episodes	Success	SR	SPL	Final Dist. (m)	Avg. Steps	VLA Kept
Full RGB-D	20	19	0.950	0.738	1.134	49.95	0.598
Zero RGB-D	20	0	0.000	0.000	8.596	7.00	1.000

**Table 15:** Template-semantic waypoint controller pilot over three AirSim Blocks seeds. Commands contain only discrete sector words rather than explicit numeric coordinates. The experiment validates a controlled waypoint interface, not open-ended language grounding.

Setting	Episodes	Success	Parse SR	Final Dist. (m)	Avg. Steps
Template-semantic waypoint controller	60	60	1.000	0.950±0.008	33.90±1.51

## Data Availability

A supporting archive accompanies this submission and contains machine-readable step records and labels for the 3385-sample dataset, representative RGB-D observations, training and evaluation scripts, the final selected checkpoints and metrics, closed-loop rollout traces used for the reported comparisons, human-readable reports, and a SHA-256 manifest. The complete raw RGB-D frame dump and local simulator intermediate files are omitted from the archive because of file-size and environment-dependency constraints; these larger artifacts will be made available by the corresponding author upon reasonable request for research verification.

## Abbreviations

The following abbreviations are used in this manuscript:

UAV	Unmanned Aerial Vehicle
VLA	Vision-Language-Action
RGB-D	Red-Green-Blue-Depth
SR	Success Rate
SPL	Success weighted by Path Length
VLM	Vision-Language Model
CPU	Central Processing Unit
CUDA	Compute Unified Device Architecture
